# Supplementary material for: Value added transformation of ubiquitous substrates into highly efficient and flexible electrodes for water splitting
Source: Nat Commun. 2018 May 22;9:2014. doi: 10.1038/s41467-018-04358-7 (PMC5964234; doi:10.1038/s41467-018-04358-7)
Supplement: Supplementary file 1 — Supplementary Information [file 41467_2018_4358_MOESM1_ESM.pdf]

Value Added Transformation of Ubiquitous Substrates into Highly Efficient and Flexible Electrodes for Water Splitting

Sahasrabudhe et al.

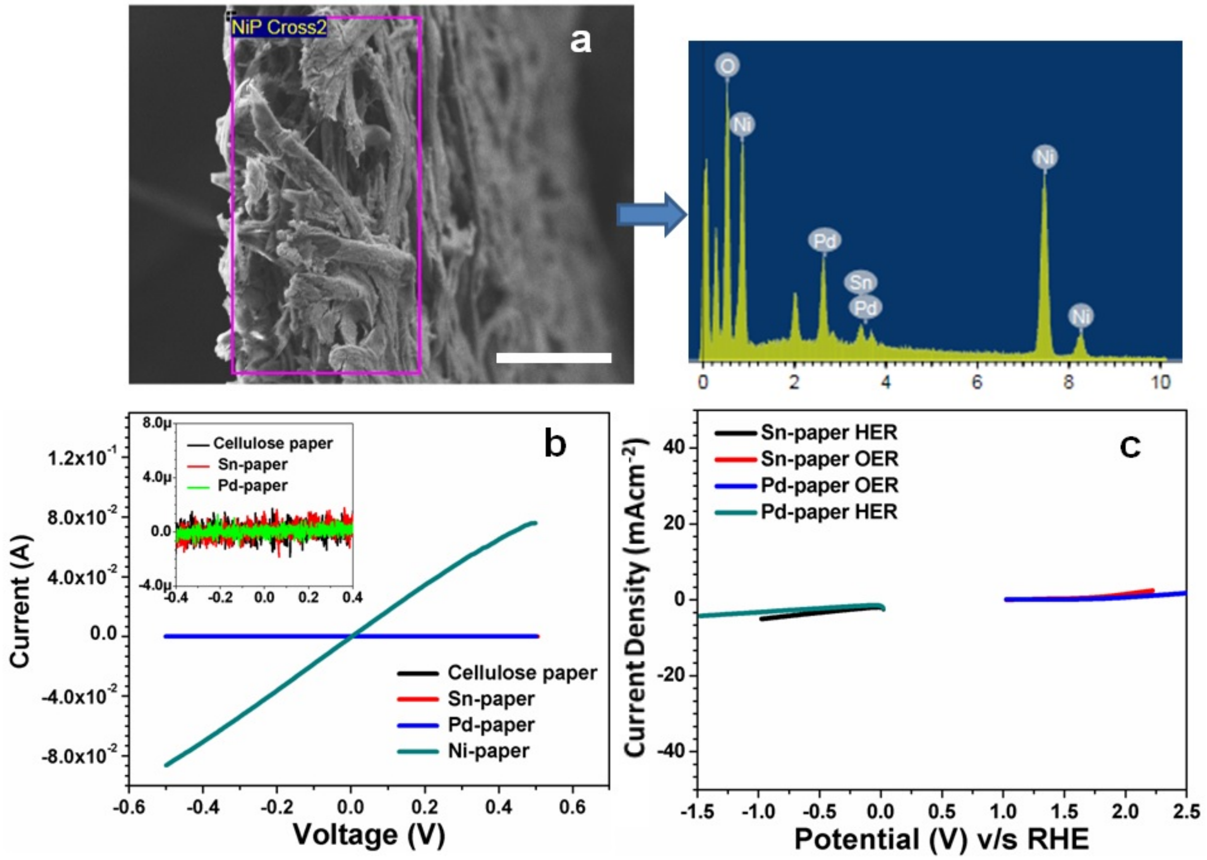

Supplementary Figure 1: Metal impurities from electroless plating and their impact on conductivity and electrocatalytic activity. **a** EDX spectrum from a selected area of the FE-SEM image of Ni-P showing the presence of Pd and Sn (scale bar 100 µm). **b** Comparison of current-voltage plots of Ni-P with cellulose paper and the control Pd-activated paper and Sn-activated paper. Inset shows the magnified view of the overlapping traces. **c** OER and HER polarization curves (iR-corrected) of Pd-activated and Sn-activated papers with a scan rate of $10 mVs^{-1}$ in 1M KOH.

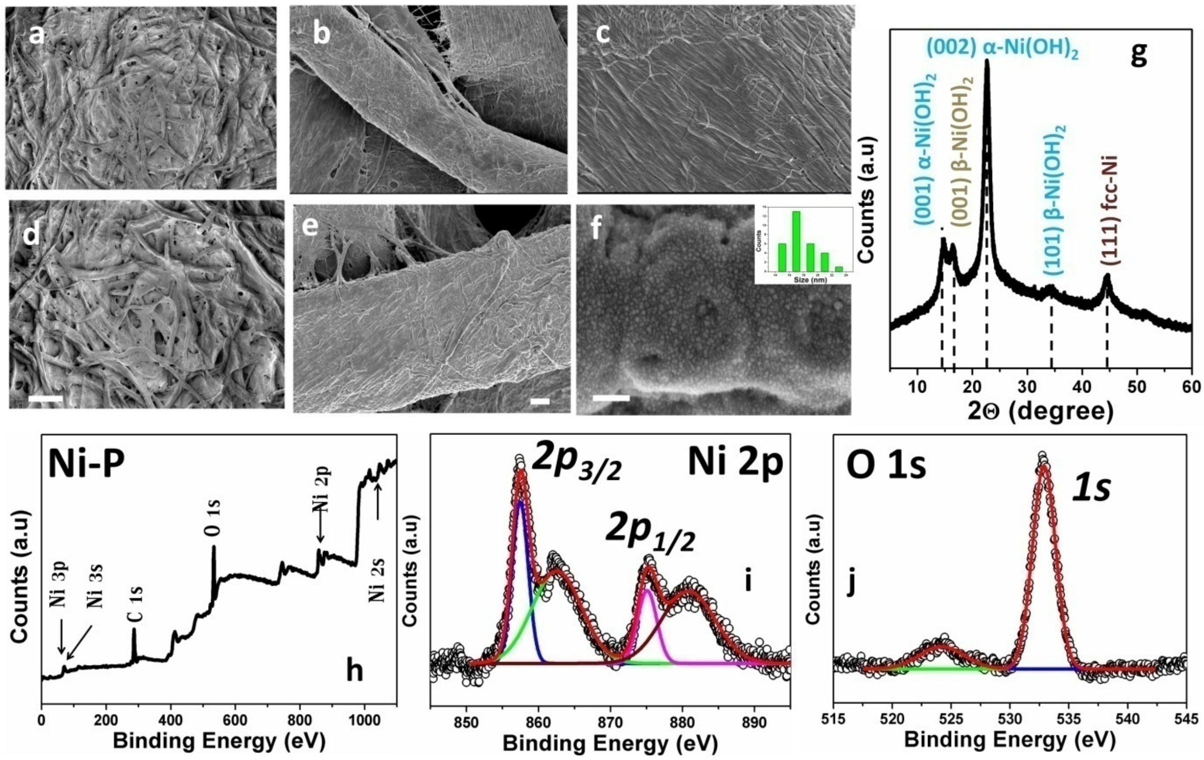

Supplementary Figure 2: FE-SEM images of bare paper and Ni-P; XRD and XPS spectra of Ni-P. **a-c** FE-SEM images at different magnifications of bare cellulose paper (scale bar 100 μm, 2 μm and 1 μm, respectively) and **d-f** Ni-P (scale bar 100 μm, 2 μm and 200 nm, respectively); **g** XRD spectrum of Ni-P; **h-j** Full survey scan and deconvoluted core level XPS spectra of Ni-P paper.

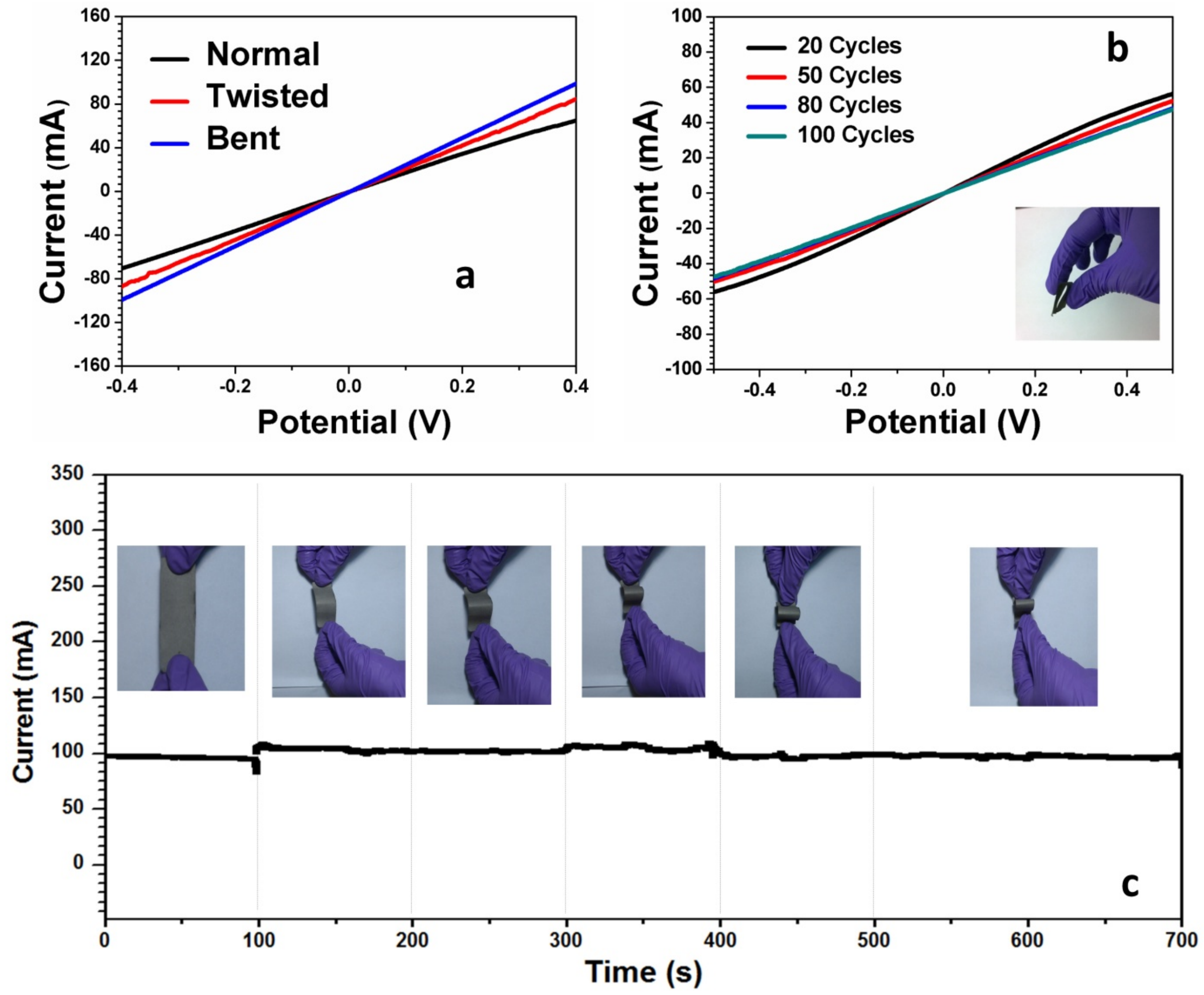

Supplementary Figure 3: Current-voltage characteristics of Ni-P under mechanical stress. **a** Current-voltage plots of Ni-P under different forms of mechanical stress; **b** after different cycles of successive bending at 0 degree, inset shows a representative digital image of the deformation; **c** Chronoamperometric response while simultaneously increasing the curvature of the fold as shown in digital images.

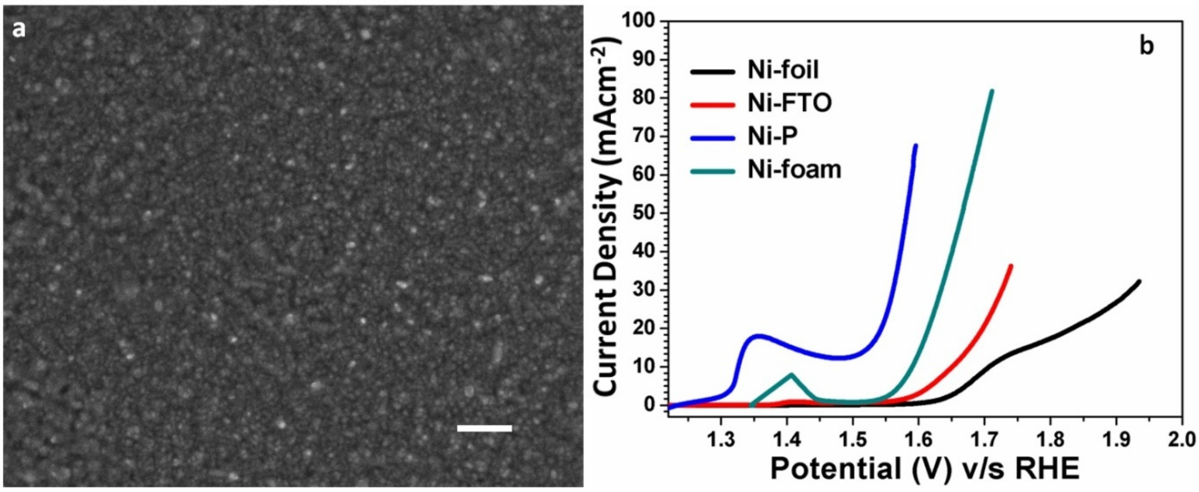

Supplementary Figure 4: FE-SEM micrographs and catalytic activity of control electrodes. **a** FE-SEM micrograph of nanostructured and non-porous Ni-FTO electrode (scale bar 200 nm). **b** OER LSV traces for different control electrodes.

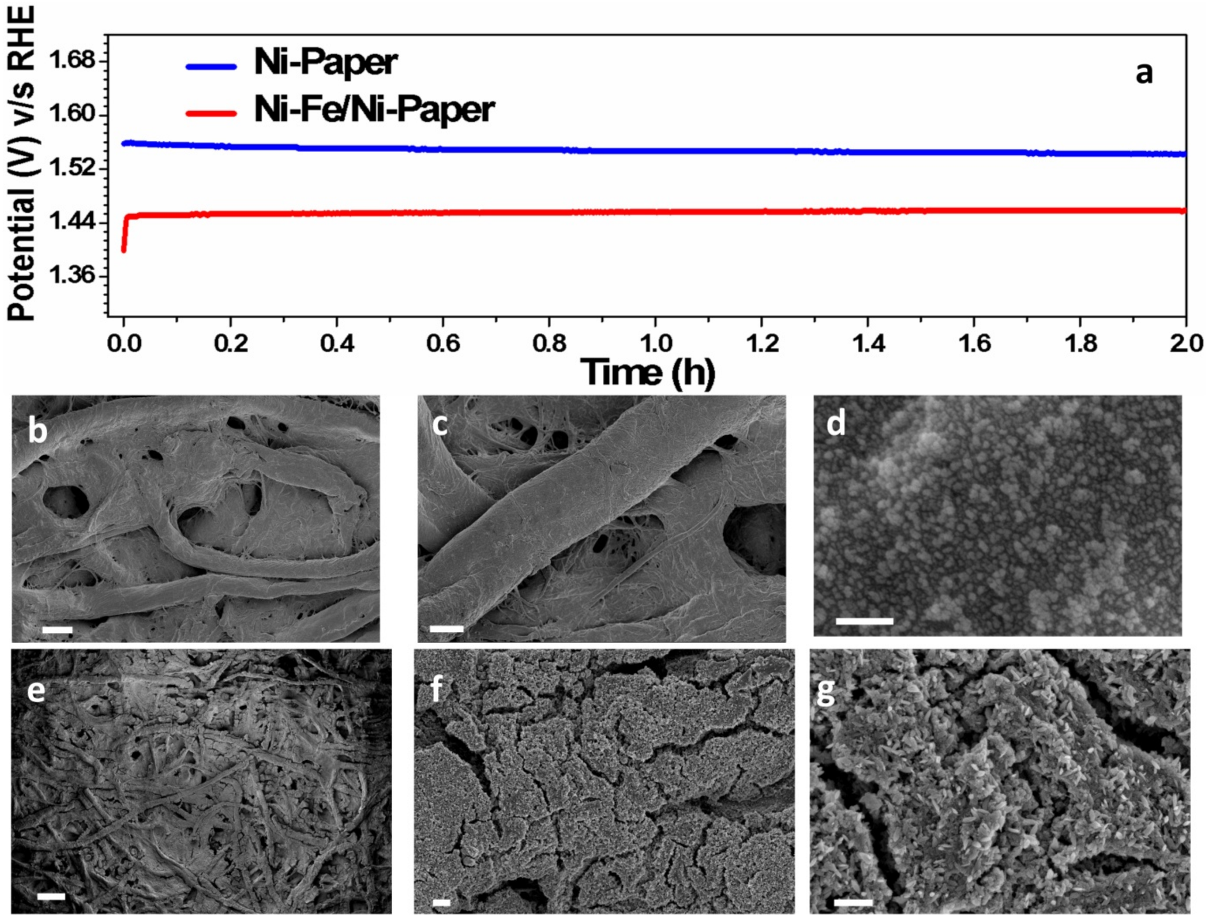

Supplementary Figure 5: Chronopotentiometric stability test and FE-SEM images of the paper electrodes after 12h bulk electrolysis under OER conditions. **a** Chronopotentiometric stability test of Ni-P and NiFe/Ni-P towards OER at a current density of $30mAcm^{-2}$ (without iR-correction); FE-SEM images at different magnifications after 12h bulk electrolysis under OER conditions for **b-d** Ni-P (scale bar 100 µm, 10 µm and 200 nm, respectively) and **e-g** NiFe/Ni-P (scale bar 100 µm, 2 µm and 1 µm, respectively).

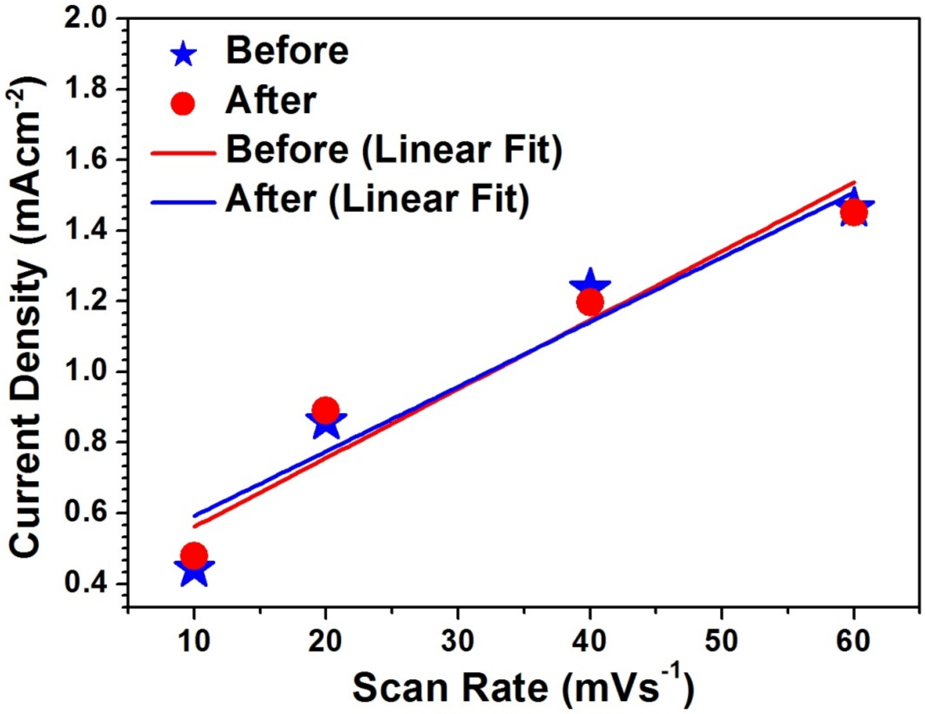

Supplementary Figure 6: ECSA of electrodes before and after stability test. Straight line fits of maximum anodic current density versus scan rate plots for NiFe/Ni-P before (circle) and after (star) 12h electrolysis.

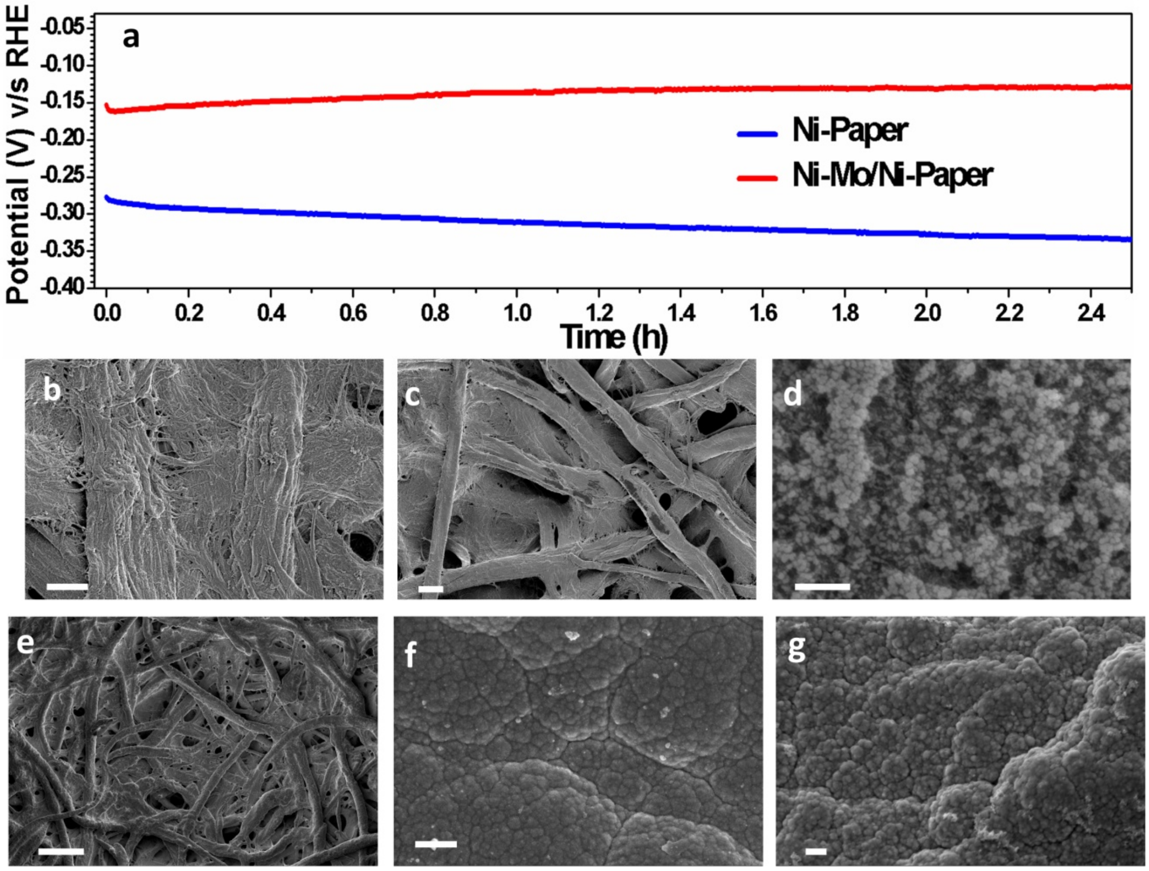

Supplementary Figure 7: Chronopotentiometric Stability Test and FE-SEM Images of the Paper Electrodes after 12h Bulk Electrolysis under HER Conditions. **a** Chronopotentiometric stability test of Ni-P and NiMo/Ni-P towards HER at a current density of -30mAcm^{-2} (without iR-correction); FE-SEM images at different magnifications after 12h bulk electrolysis under HER conditions for **b-d** Ni-P (scale bar 100 µm, 2 µm and 200 nm, respectively) and **e-g** NiMo/Ni-P (scale bar 100 µm,1 µm and 500 nm, respectively).

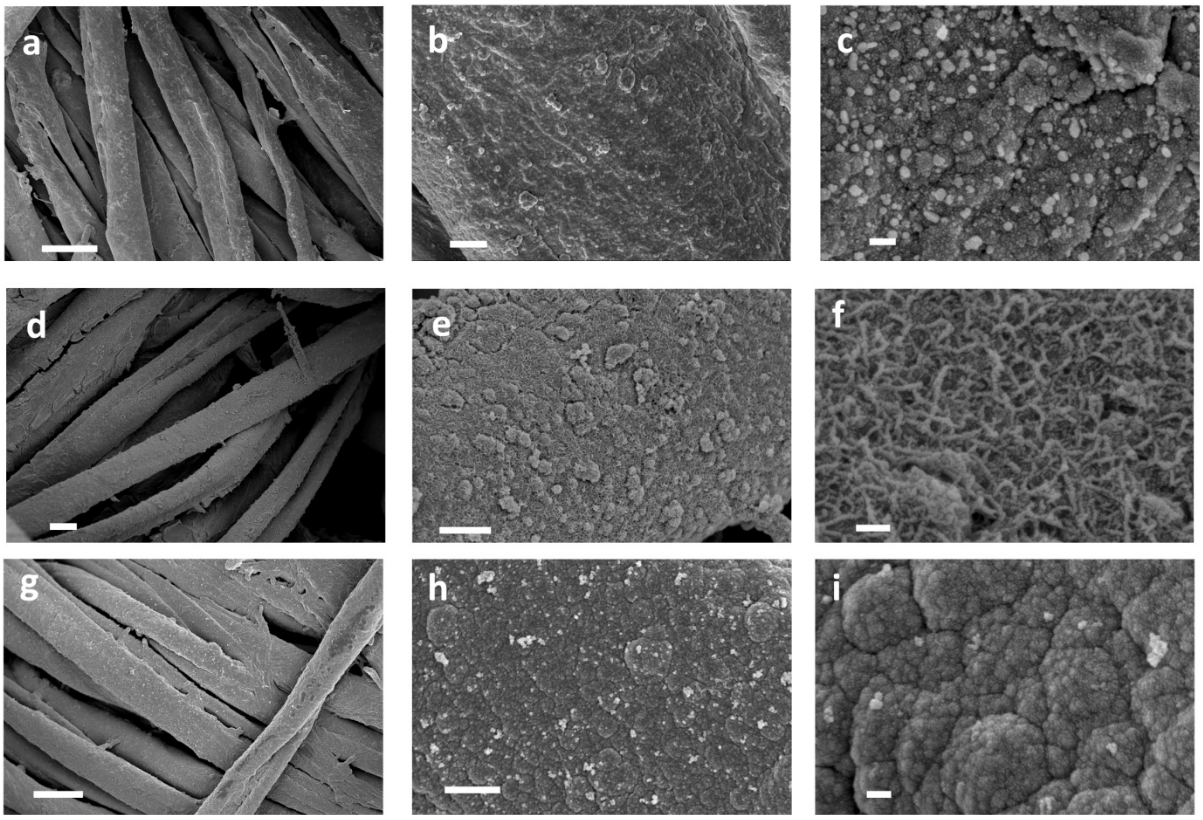

Supplementary Figure 8: FE-SEM images at different magnifications. **a-c** Ni-CF (scale bar 20 μm. 2 μm and 200 nm, respectively) **d-f** NiFe/Ni-CF (scale bar 10 μm, 2 μm and 200 nm, respectively), **g-i** NiMo/Ni-CF (scale bar 20 μm, 2 μm and 200 nm, respectively).

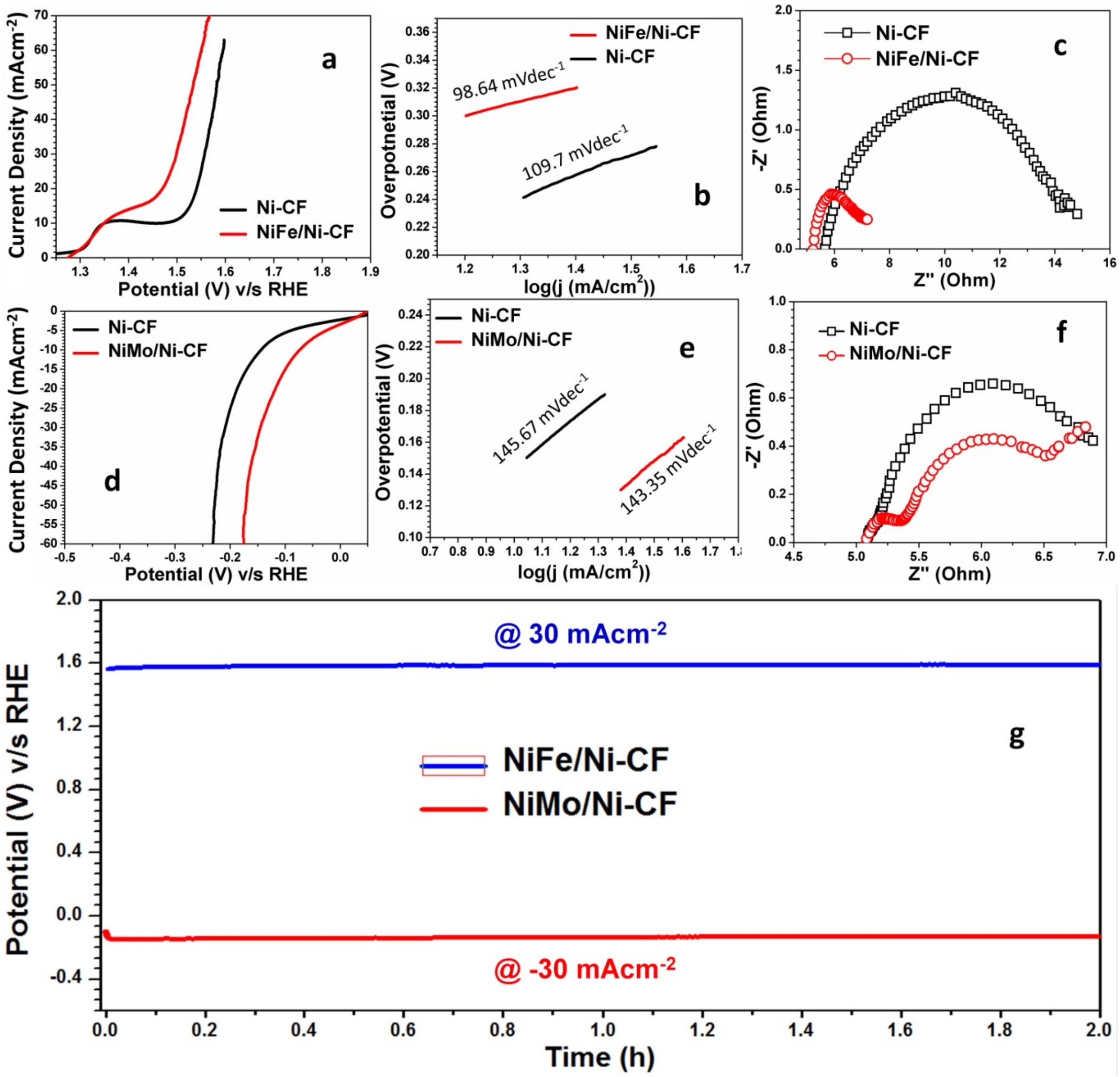

Supplementary Figure 9: OER and HER electrocatalytic activity of NiFe and NiMo modified flexible cloth electrodes, respectively. **a** LSV; **b** Tafel analysis and **c** EIS spectrum of Ni-CF and NiFe/Ni-CF OER anodes; **d** LSV; **e** Tafel analysis and **f** EIS spectrum of Ni-CF and NiMo/Ni-CF HER cathodes; **g** Chronopotentiometric stability test of NiFe/Ni-CF and NiMo/Ni-CF electrodes.

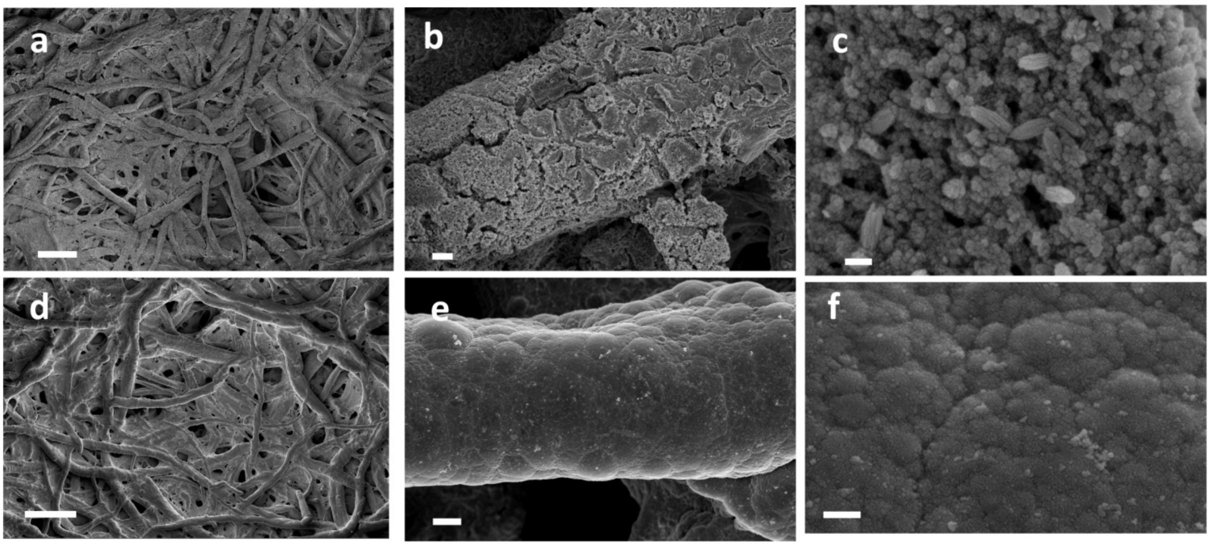

Supplementary Figure 10: FE-SEM images at different magnifications after 20h bulk electrolysis of NiFe-NiMo paper electrolyzer. **a-c** NiFe/Ni-P anode (scale bar 100 μm, 2 μm and 200 nm, respectively) and **d-f** NiMo/Ni-P cathode (scale bar 100 μm, 2 μm and 200 nm, respectively).

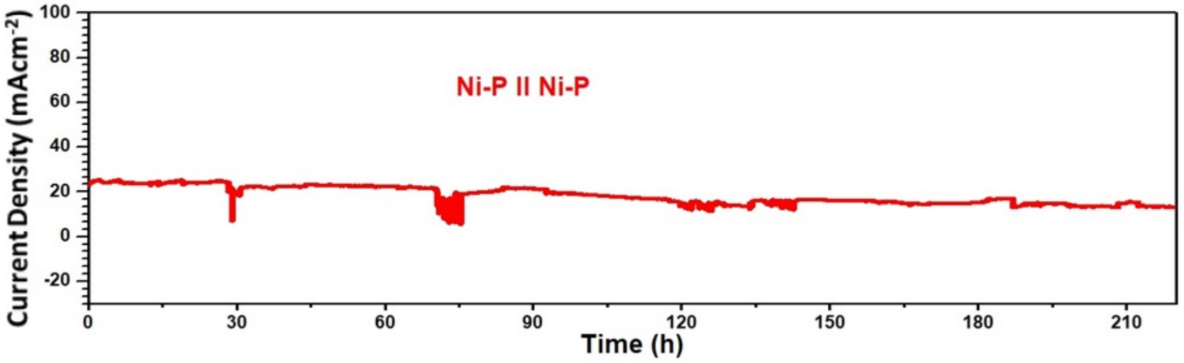

Supplementary Figure 11: Chronoamperometric stability test of bi-Ni-P electrolyzer in 1M KOH at 1.8V for >200h.

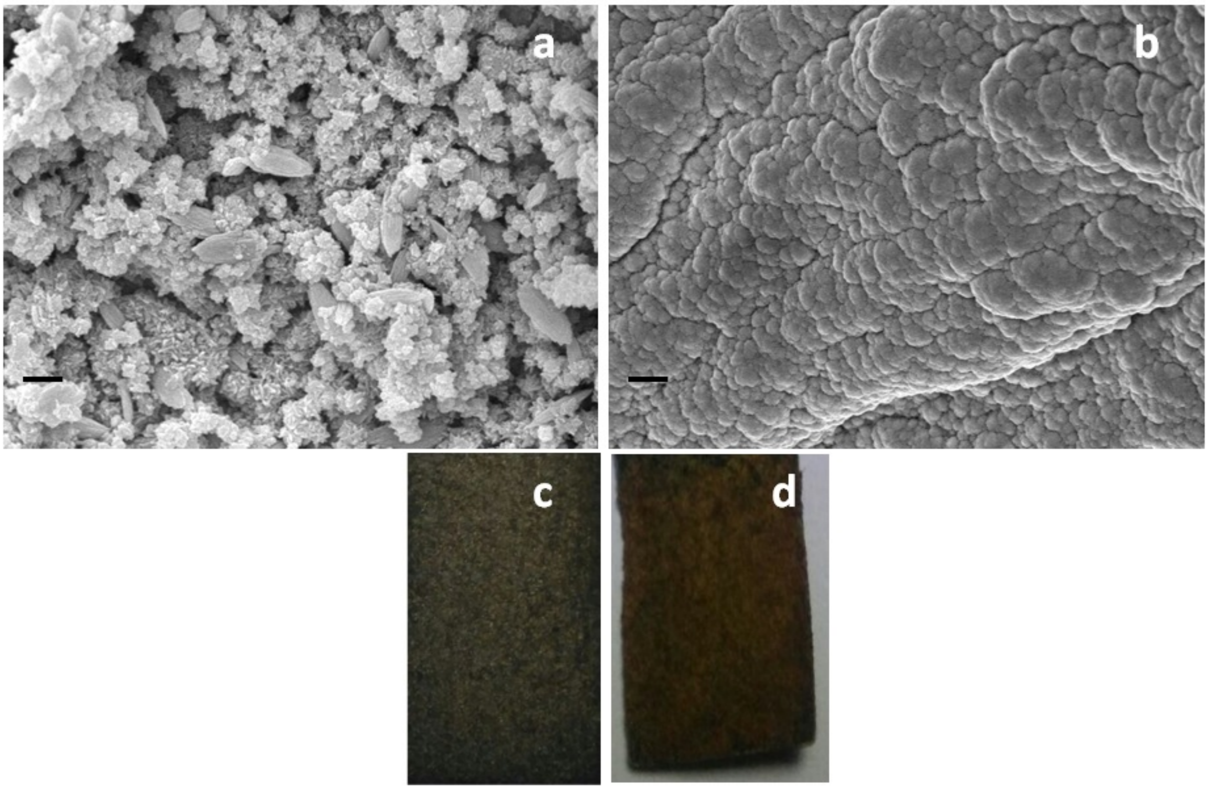

Supplementary Figure 12: Structural Integrity after 12h Electrolysis in 10M KOH. **a, b** FE-SEM (scale bar 200 nm) and **c, d** digital images of NiFe/Ni-P and NiMo/Ni-P electrodes, respectively after 12h electrolysis under extreme conditions in 10M KOH.

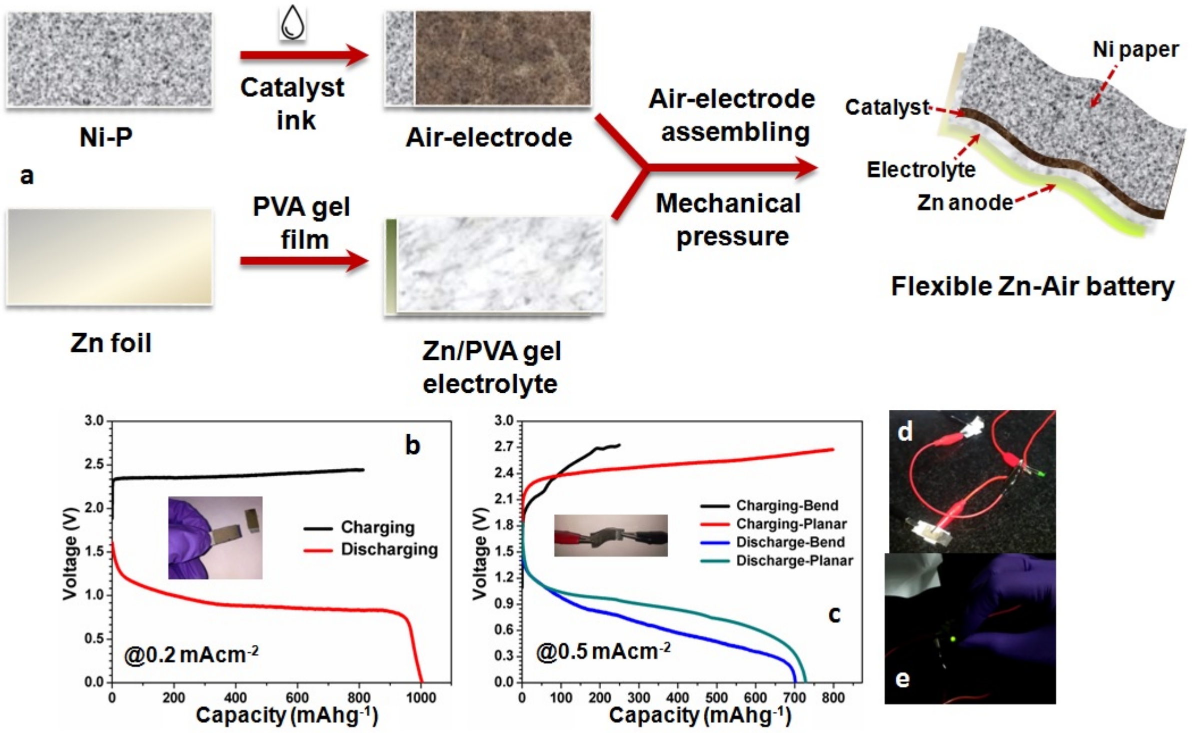

Supplementary Figure 13: Zn-Air battery prototype using paper-electrodes. **a** Schematic of fabrication procedure of the wearable Zn-air battery. **b** Constant load charge-discharge performance of a planar Zn-air battery at 0.2mAcm^{-2}, and **c** a bent v/s planar Zn-air battery at 0.5mAcm^{-2}. Insets show digital images of the flexible batteries. **d** Two Zn-air batteries connected in series to light-up a commercial LED.

Supplementary Table 1: Electrochemical parameters of different OER catalysts. Abbreviations: j_0 = exchange current density, R_s = series resistance, and R_{ct} = charge transfer resistance.

**Catalyst**	**Onset Potential[a][b] (V v/s RHE)**	**η^{50}[b] (V)**	**Tafel slope ($mVdec^{-1}$)**	**j_0 ($\times 10^{-6}$ $mAcm^{-2}$)**	**R_s (Ω)**	**R_{ct} (Ω)**
Ni-P	1.51±0.02	0.35±0.03	78.37	1.73	4.22	1.96
NiFe/Ni-P	1.44±0.03	0.24±0.02	29.66	5.49	3.81	0.44
IrO_2	1.49±0.02	0.39±0.01	54.81	28.9	N.A	NA
Ni-foam	1.54±0.04	0.44±0.02	81.77	2.13	4.17	1.78
Ni-CF	1.52±0.03	0.35±0.04	98.64	1.94	5.23	9.38
NiFe/Ni-CF	1.43±0.02	0.30±0.03	109.7	0.017	5.47	2.19

[a] Calculated from the tangent to the OER wave
[b]Average and standard deviation of n = 3 electrodes

Supplementary Table 2: Comparison of OER performance of paper-electrodes with highly active electrocatalysts under alkaline conditions

**Catalyst**	**η^a (mV)**	**Tafel Slope (mV dec^{-1})**	**Electrolyte**	**Substrate**	**Reference**
N-doped graphene-CoO	340	71	1 M KOH	Glassy Carbon	1
Co_3O_4/N-doped graphene	310	67	1 M KOH	Ni-foam	2
$Zn_xCo_{3-x}O_4$ NRA	320	51	1 M KOH	Ti foil	3
$Ni_xCo_{3-x}O_4$	370	59-64	1 M NaOH	Ti Foil	4
Ni substituted Co_3O_4 NRA	370	65-74	1 M NaOH	Ni-foam	5
Ni-P	300	64	1 M KOH	Glassy Carbon	6
$Ni(OH)_2$	360	111	1 M KOH	Glassy Carbon	6
NiO	430	81	1 M KOH	Glassy Carbon	6
Ni_2P nanoparticles	290	59	1 M KOH	Glassy Carbon	7
Ni_2P nanowires	330	47	1 M KOH	Glassy Carbon	7
Amorphous NiCo binary oxide	325	39	1 M NaOH	Gold substrate	8
Ni-Co hierarchical nanosheets	340	51	1 M NaOH	FTO	9
CoMn-LDH nanosheets	324	43	1 M KOH	Carbon fibre paper	10
5.9 nm Co_3O_4	328	47	1 M KOH	Glassy Carbon	11
Ni-P	352[b]	78	1 M KOH	Cellulose Paper	This Work
NiFe/Ni-P	241[b]	29	1 M KOH	Cellulose Paper	This Work

[a] Overpotential at 10mAcm^{-2} unless otherwise stated
[b] Overpotential at 50mAcm^{-2}

Supplementary Table 3: Electrochemical parameters of different HER catalysts. Abbreviations: j_0 = exchange current density, R_s = series resistance, and R_{ct} = charge transfer resistance.

**Catalyst**	**Onset Potential[a][b] (V v/s RHE)**	**$\eta^{10[b]}$ (V)**	**Tafel slope ($mVdec^{-1}$)**	**j_0 ($\times 10^{-4}\ mAcm^{-2}$)**	**R_s (Ω)**	**R_{ct} (Ω)**
Ni-P	-0.09±0.02	0.12±0.03	85.12	0.30	3.34	1.68
NiMo/Ni-P	-0.01±0.03	0.31±0.02	60.85	2.04	2.84	0.77
Pt/C	-0.01±0.01	0.15±0.01	28.33	2.97	N.A	N.A
Ni-foam	-0.12±0.02	0.33±0.01	212.02	0.75	4.73	83.7
Ni-CF	-0.10±0.03	0.14±0.04	145.67	0.9	5.09	2.53
NiMo/Ni-CF	-0.05±0.03	0.73±0.03	143.35	3.01	5.09	1.97

[a]Calculated from the tangent to the HER wave
[b]Average and standard deviation of n = 3 electrodes

Supplementary Table 4: Comparison of HER performance of paper-electrodes with highly active electrocatalysts in alkaline medium

**Catalyst**	**η[a] (mV)**	**Tafel Slope (mV dec^{-1})**	**Electrolyte**	**Substrate**	**Reference**
CoP-mesoporous NRA	54	51	1M KOH	Ni foam	12
Porous MoO_2/Ni-foam	25	41	1M KOH	Ni-foam	13
$NiCo_2S_4$ NW/Ni-foam	210	58	1M KOH	Ni-foam	14
MoO_x/Ni_3S_2-Ni-foam	106	90	1M KOH	Ni-foam	15
NiFe/$NiCo_2O_4$/NF	105	88	1M KOH	Ni-foam	16
CP@Ni-P	117	85	1M KOH	Carbon fibre paper	17
Li-induced TMO/CFP	88	150	1M KOH	Carbon fibre paper	18
Ni_3FeN-NPs	158	49	1M KOH	Ni-foam	19
np-$(Co_{0.52}Fe_{0.48})_2P$	64	45	1M KOH	Self-Supported	20
CoP/CC	67	129	1M KOH	Carbon cloth	21
CoP Film	94	42	1M KOH	Cu Foil	22
FeP NAs/CC	218	146	1M KOH	Carbon Cloth	23
Co-Co_3O_4	100	44	1M KOH	Ni-foam	24
NiMoN	109	95	1M KOH	Carbon Cloth	25
MoON	146	101	1M KOH	Carbon Cloth	25
Ni_3N	208	113	1M KOH	Carbon Cloth	25
CP/CT/Co-S	190	131	1M KOH	Carbon fibre paper	26
MoP	140	48	1M KOH	Glassy Carbon	27
Mo_2N	353	108	1M KOH	Glassy Carbon	28
MoC_x	151	59	1M KOH	Glassy Carbon	29
NiO/Ni-CNT	80	82	1M KOH	Ni-foam	30
MoB	220	59	1M KOH	Glassy carbon	31
$Ni_{2/3}Fe_{2/3}$-rGO	560	210	1M KOH	Ni-foam	32
Ni-P	128	85	1M KOH	Cellulose Paper	This Work
NiMo/Ni-P	31.6	60	1M KOH	Cellulose Paper	This Work

[a] Overpotential at -10mAcm^{-2}

Supplementary Table 5: Electrochemical parameters for intrinsic activity of NiFe and NiMo.

**Catalyst**	**C_{dl} ($mFcm^{-2}$)**	**σ**	**$j_0 \sigma^{-1}$ @ $\eta = 0V$ (μAcm^{-2})**	**$J \sigma^{-1}$ ($mAcm^{-2}$)**
Ni-P (OER)	30.78	769.5	2.24×10^{-3}	0.015[a]
NiFe/Ni-P	62.76	1569	3.49×10^{-3}	0.063[a]
Ni-P (HER)	30.78	769.5	0.38	-0.0068[b]
NiMo/Ni-P	3.6	90	22.66	-1.11[b]

[a] @ $\eta = 250mV$
[b] @ $\eta = 100mV$

Supplementary Table 6: Performance comparison of recently reported non-noble electrocatalysts for overall water splitting under alkaline conditions.

**Electrolyzer**		**E@10 mAcm^{-2}(V)**	**Electrolyte**	**Reference**
**Anode**	**Cathode**			
CP/CTs/Co-S	CP/CTs/Co-S	1.74	1M KOH	26
NiFe-LDH/Ni-F	NiFe-LDH/Ni-F	1.70	1M NaOH	33
NiO/Ni-CNT/Ni-F	NiFe/Ni-F	1.5	1M KOH	30
CoO_x/NC	CoO_x/NC	1.62	1M KOH	34
$Ni_{0.33}Co_{0.67}S_2$/Ti-foil	$NiCo_2O_4$/Ti-foil	1.73	1M KOH	35
Co-P MNA/Ni-F	Co-P MNA/Ni-F	1.62	1M KOH	12
Ni-P@CP	Ni-P@CP	1.63	1M KOH	17
Li-induced $NiFeO_x$	Li-induced $NiFeO_x$	1.51	1M KOH	18
MoO_x/Ni_3S_2-NF	MoO_x/Ni_3S_2-NF	1.45	1M KOH	15
$NiCo_2S_4$/NF	$NiCo_2S_4$/NF	1.63	1M KOH	14
Porous MoO_2/NF	Porous MoO_2/NF	1.53	1M KOH	13
NiFe/$NiCo_2O_4$/NF	NiFe/$NiCo_2O_4$/NF	1.67	1M KOH	16
NiSe/NF	NiSe/NF	1.63	1M KOH	36
Ni_5P_4/Ni-foil	Ni_5P_4/Ni-foil	<1.7	1M KOH	37
Ni_2P	Ni_2P	1.63	1M KOH	7
Co-P/Cu foil	Co-P/Cu foil	1.64	1M KOH	22
np-$(Co_{0.52}Fe_{0.48})_2P$	np-$(Co_{0.52}Fe_{0.48})_2P$	1.53	1M KOH	20
CoMnO@CN on NF	CoMnO@CN on NF	1.50	1M KOH	38
EG/$Co_{0.85}$Se/NiFe	EG/$Co_{0.85}$Se/Ni Fe	1.67	1M KOH	39
$NiCo_2S_4$/CC	$NiCo_2S_4$/CC	1.68	1M KOH	40
Ni_2P/NiO_x	Ni_2P/NiO_x	1.63	1M KOH	7
$Ni(OH)_2/NiSe_2$	$Ni(OH)_2/NiSe_2$	1.78	1M KOH	41
ONPPGC/OCC	ONPPGC/OCC	1.66	1M KOH	42
Ni-P	Ni-P	1.69	1M KOH	This Work
NiFe/Ni-P	NiMo/Ni-P	1.51	1M KOH	This Work
NiFe/Ni-P	NiMo/Ni-P	1.49	10M KOH	This Work

Supplementary Discussion 1: Role of Porous-Nanostructured Interfaces in Paper Electrodes - Structure Property Correlation

To further our understanding of the underpinning mechanistic factors and develop a structure-function correlation for paper electrodes, we perform a comparative electrocatalytic study with four control systems that have well defined structural features of porosity and nanostructure. To begin with, we hypothesize that for Ni-paper based electrodes, the nanostructured nature of Ni as well as the hierarchical porosity imparted by the underlying paper template are responsible for their impressive electrocatalytic activity. To test this hypothesis we first compare the OER performances of two porous electrodes, namely unmodified Ni-P and commercial Ni-foam. From the corresponding LSV traces (Supplementary Figure 3) it is apparent that Ni-P requires much lower overpotential than Ni-foam towards OER under otherwise identical conditions. This interesting observation provides an important physical insight. Although both Ni-foam and Ni-P are highly porous substrates, Ni-foam is composed of bulk Ni metal which is in stark contrast with the nanoparticulate Ni of Ni-paper (average size of ~23nm as observed from electron microscopy). Thus, the improved activity of Ni-P over Ni-foam can be attributed to the nanostructured nature of Ni-P interface. Next, we compare the activity of Ni-P with a nonporous but nanostructured electrode to understand the role of porosity. To this end a non-porous control electrode was prepared by electroless plating of Ni nanoparticle thin film on FTO-glass (abbreviated as Ni-FTO) using a process identical to that used for paper substrates. The FE-SEM micrograph of the as prepared Ni-FTO (Supplementary Figure 3) reveals the formation of a uniform thin film composed of spherical Ni nanoparticles with a high degree of homogeneity and absence of any pores, unlike those seen for Ni-papers. The electrochemical activities of Ni-P and Ni-FTO towards OER under identical conditions indicate the superior performance of Ni-P. Thus although both Ni-P and Ni-FTO are composed of nanostructured Ni, the former, the hierarchical porous structure in the former offers higher electrochemically active surface and hence provides improved mass transport kinetics. Finally to confirm these conclusions OER activity of commercial Ni-foil was measured. Commercial Ni-foil was chosen as the control electrode as it provides both non-porous and non-nanostructured electrochemical interface and thus serves as a perfect choice to test our hypothesis. Clearly Ni-foil with bulk Ni and without any hierarchical porosity was least active towards OER (Supplementary Figure 3), thereby validating our hypothesis. To

sum it all, we conclude that two structural features that are unique to Ni-paper electrodes, namely i) nanostructured nature of Ni and; ii) porous nature of the underlying paper substrate act in a synergistic fashion to lend exceptional electrocatalytic activities to Ni-paper electrodes. While Ni nanoparticles with large surface-to-volume ratio have abundance of exposed active sites for catalysis, the interweaving pores can render faster mass-transport kinetics. Thus by a careful choice of control electrodes, this comparative study establishes the underpinning structure-function correlation in Ni-paper electrodes.

Supplementary Discussion 2: Use of Tafel Analysis for Comparing the Electrocatalysts

In the present work an apparent contradiction was observed as the exchange current density of IrO_2 was found to be higher than that of NiFe/Ni-P electrode, inspite of lower overpotentials observed for the latter. We would like to stress that similar observations were made after repeated measurements which rules out serendipity and artefacts. Here we would like to add a word of caution while drawing conclusions about the electrochemical activity of different electrocatalysts using the Tafel analysis. Use of Tafel analysis to compare electrocatalysts can often be misleading particularly for multi-step, multi-electron reactions. The two main quantities of interest extracted from a Tafel plot are the exchange current density (j_0) and the Tafel slope. While j_0 is a measure of intrinsic kinetic activity of an electrocatalyst, Tafel slope is a descriptor of the reaction mechanism. It is known that j_0 derived from Tafel analysis tends to have significant errors and hence is not a reliable parameter of merit while comparing activities of different catalysts[43]. This also explains the apparent contradiction between activities and j_0 values for IrO_2 and NiFe/Ni-P electrode observed in this work. Similarly Tafel slopes can be deceptive mainly because of their potential dependent nature particularly for multi-step electrochemical reactions. This can often be seen in the form of two or more Tafel slopes in different overpotential intervals which can in-turn be attributed to potential dependent changes in the catalytic mechanism. However, the data obtained from Tafel analysis is still retained in this work for the sake of completeness and consistency with prior literature in this field.

Supplementary Discussion 3: Effect of Morphology on Electrochemical Stability of NiFe/Ni-P Electrodes

We would like to note an important aspect point about performance of OER electrodes after 12h stability tests and the accompanying morphology change. From the FE-SEM micrographs in Supplementary Figure 4 it is quite apparent that the initial vertically aligned flake type morphology of NiFe gives way to a rice-grain type structure after 12h electrolysis. Surprisingly enough this morphology transformation has a minimal effect on catalytic activity of NiFe/Ni-P as observed from similar η^{50} values before and after stability tests (inset Figure 5e, main text). Moreover, the stable current outputs delivered by NiFe/Ni-P in a two-electrode device for more than 200h of continuous operation (Figure 9f, main text) also suggest sustained activity post morphology alteration. We would like to state that similar results were obtained on repeated trials at varying current densities for 12h chronoamperometric tests. Careful analysis of FE-SEM images post stability tests, however, reveals presence of a porous microstructure for NiFe. To investigate this aspect further, the electrochemically active surface area (ECSA) of NiFe/Ni-P electrode was compared before and after 12h stability tests. The similar slopes obtained for the plot of current density v/s scan rate (Supplementary Figure 5) indeed demonstrates that the ECSA of NiFe/Ni-P post stability is almost identical to that before. This helps us to rationalize the observations. We can conclude that although the morphology of NiFe is physically transformed over the course of long term electrolysis, the active area that contributes to the electrochemical current still remains the same and hence is responsible for stable electrode performance.

Supplementary Discussion 4: Faradic Efficiency of bare Ni-P electrodes

Faradic efficiency for bare Ni-P electrodes towards both OER and HER was determined using a custom built eudiometry set-up. Interestingly the Ni-P electrodes showed a near perfect Faradic efficiency of 99.2% for hydrogen evolution as determined from comparing the theoretical moles of gas evolved at different time intervals with the experimentally measured quantities (using Faraday's law and ideal gas approximation). This near unity value strongly suggests complete consumption of charge to produce H_2 without any allied parasitic reactions and also compares favourably with the performance of NiMo/Ni-P electrode (99.4%). On the other hand the Faradic efficiency of Ni-P towards OER (86.7%) was found to be lower than that for NiFe/Ni-P (97.3%) suggesting an incomplete conversion of charge into O_2 for unmodified Ni-P. This lower Faradic efficiency for bare Ni-P can be attributed to the scavenging of charge by a competing $2e^-$ oxidation of Ni^0 into Ni^{2+} which is kinetically much more facile than the sluggish $4e^-$ water oxidation reaction. This also highlights the catalytic role of the NiFe top layer in NiFe/Ni-P electrodes, where NiFe improves the charge transfer kinetics with OH^- in the electrolyte and thereby out-competes the Ni oxidation pathway. This eventually results in an effective generation of oxygen with a high Faradic efficiency for NiFe/Ni-P as compared to bare Ni-P electrodes.

Supplementary Discussion 5: Substrate Scope

In the discussion above it was shown how a simple piece of cellulose paper can be transformed into high performing, robust and flexible electrodes for water splitting. To take this further and to understand the generalizability of our approach to other commonly available flexible substrates, the same electrodes were fabricated on a piece of cotton fabric. Using an identical method as that used for cellulose paper, metallic Ni was deposited onto the cotton fabric (abbreviated as Ni-CF henceforth). The low magnification FE-SEM images of Ni-CF show an obvious open framework of intertwined fibres which indicates selective deposition of metallic Ni only along the cellulose strands, while higher resolution images revealed the nanoparticulate nature with an average size of Ni^0 NPs estimated to be ~32nm (Supplementary Figure 8a-c). The active catalysts of NiFe and NiMo were further electrodeposited onto Ni-CF (abbreviated as NiFe/Ni-CF and NiMo/Ni-CF, respectively). The FE-SEM images of these electrodes showed a vertical array of NiFe nanoflakes with a macropore size of ~50nm (Supplementary Figure 8d-f) for NiFe/Ni-CF while large spherical aggregates of bimetallic NiMo uniformly covered the surface while also preserving the basic 3-D architecture of the underlying cotton fabric in case of NiMo/Ni-CF (Supplementary Figure 8g-i). The unambiguous similarity in the microstructures of Ni-CF, NiFe/Ni-CF, NiMo/Ni-CF and their respective paper-based counterparts establishes the wide-applicability and generalization of our method for fabricating flexible electrodes on common substrates for water splitting. The catalytic activity of as prepared cotton-based electrodes was then tested towards OER and HER half reactions under alkaline conditions and the relevant catalytic parameters are summarized in Supplementary Table 1 and Supplementary 3. The catalyst modified anode NiFe/Ni-CF (η^{50} = 290mV) shows improved OER activity than the bare Ni-CF (η^{50} = 360mV) with lower onset potential and smaller η^{50} value (Supplementary Figure 9a), which also outperforms the benchmark IrO_2 and other state-of-the-art non-noble catalysts (Supplementary Table 1). These observations are also supported by lower Tafel slope of 98.64mVdec^{-1} (Supplementary Figure 9b) and higher exchange current density of 1.94× 10^{-6} mAcm^{-2} for NiFe/Ni-CF as compared to those for Ni-CF (b = 109.7 mVdec^{-1}, j_0 = 0.017× 10^{-6} mAcm^{-2}). The R_{ct} value extracted from EIS analysis (Supplementary Figure 9c) also reveals the ease of interfacial charge transfer for NiFe/Ni-CF (R_{ct} = 2.2 Ω) over the bare Ni-CF electrode (R_{ct} = 9.1 Ω). Similarly, NiMo/Ni-CF showed improved HER voltammograms (Supplementary Figure 9d) with η^{10} value of 77mV as compared to that of 151mV for Ni-CF.

Notably NiMo/Ni-CF even shows better activity than other recently reported non-noble catalysts (Supplementary Table 3). As expected the excellent HER activity of NiMo/Ni-CF was accompanied by higher exchange current density of 3.01mAcm^{-2} and a smaller R_{ct} value of 2.1 Ω as compared to those for the bare Ni-CF substrate (j_0 = 0.9mAcm^{-2}, R_{ct} = 2.4 Ω) (Supplementary Figure 9e-f). It must be noted that although NiFe/Ni-CF and NiMo/Ni-CF show excellent performance towards OER and HER, their activities are inferior to paper-based electrodes of NiFe/Ni-P and NiMo/Ni-P, respectively. This difference is believed to originate from mass transport limitations for CF based substrates as the product gas bubbles tend to stick to the electrode and grow bigger in size before being released off. This sluggish process is in direct contrast with rapid nucleation of tiny bubbles and their fast dissipation from the surface for paper-based anode and cathode. Besides, the slightly low conductivity of Ni-CF (~6 Ωsq^{-1}) than Ni-P (4.2 Ωsq^{-1}) and larger sized Ni NPs on Ni-CF (~32nm) as compared to Ni-P (~23nm) are believed to contribute to the poorer catalytic performance by resisting the transport of charges through the Ni-CF based electrodes as well as across the electrode/electrolyte interface, and diminishing the exposed active sites, respectively. In spite of this, it is worth highlighting that NiFe/Ni-CF and NiMo/Ni-CF perform much more efficiently than many reported non-noble catalysts. The stability of CF electrodes was also tested for long term applications using chronopotentiometry (without iR correction). Supplementary Figure 9g shows that both NiFe/Ni-CF and NiMo/Ni-CF operate at constant overpotentials of 350 and 170mV, respectively to deliver a current density of 30mAcm^{-2} over a period of at least 2h without any loss of activity.

Supplementary Discussion 6: Catalyst ink loaded Ni-P as Gas Diffusion Electrode in Wearable Zn-air Battery

The Ni-P was employed as the gas diffusion electrode (GDE) in a flexible solid state rechargeable zinc air battery.

Zn-air battery fabrication: The fabrication of the wearable Zn-air battery is demonstrated in the schematic (Supplementary Figure 13a). A layer-by-layer assembly was employed with Zn foil (thickness 0.25 mm, Alfa Aesar) as anode, PVA gel film soaked in 6M KOH as electrolyte and Ni-P coated with Co_3O_4 catalyst ink as GDE. To generate absolute contact between the layers, this assembly was mechanically pressed for 30min. Over an active area of 1cm × 1cm, the catalyst loading at GDE was 1.02mg.

Zn-air battery performance: Battery performance was analyzed with a constant load charging and discharging process. An impressive specific capacity of 1003 $mAhg^{-1}$ was obtained for our solid state battery at a modest discharging current of $0.2mAcm^{-2}$, which delivered a steady state output voltage of 0.9V (Supplementary Figure 13b). The effect of device flexibility on its performance was also studied by charging and discharging the battery in a bent state at $0.5mAcm^{-2}$ (Supplementary Figure 13c). Expectedly even under mechanical stress the battery delivered a specific capacity of 700 $mAhg^{-1}$, which corresponds to ~97% of the specific capacity value obtained in planar cell (726 $mAhg^{-1}$). Further work is underway to test battery performance in a controlled and variable mechanical loading. To show the practicality of this device we also demonstrate that 2 Zn-air batteries connected in series successfully light-up a commercial LED (Supplementary Figure 13d, e). These preliminary un-optimized results are indeed very promising and further work is currently underway for systematic improvements.

Supplementary References

1. Mao, S., Wen, Z., Huang, T., Hou, Y., Chen, J. High-Performance bi-functional electrocatalysts of 3d crumpled graphene–cobalt oxide nanohybrids for oxygen reduction and evolution reactions. *Energy Environ. Sci.* **7**, 609 (2014).

2. Liang, Y. Y., Li, Y. G., Wang, H. L., Zhou, J. G., Wang, J., Regier, T., Dai, H. Co_3O_4 nanocrystals on graphene as a synergistic catalyst for oxygen reduction reaction. *Nature Mater.* **10**, 780 (2011).

3. Liu, X., Chang, Z., Luo, L., Xu, T., Lei, X., Liu, J., Sun, X. Hierarchical $Zn_xCo_{3x}O_4$ nanoarrays with high activity for electrocatalytic oxygen evolution. *Chem. Mater.* **26**, 1889 (2014).

4. Li, Y., Hasin, P., Wu, Y. $Ni_xCo_{3-x}O_4$ nanowire arrays for electrocatalytic oxygen evolution. *Adv. Mater.* **22**, 1926 (2010).

5. Lu, B., Cao, D., Wang, P., Wang, G., Gao, Y. Oxygen evolution reaction on Ni-substituted Co_3O_4 nanowires array electrodes. *Int. J. Hydrogen Energy* **36**, 72 (2011).

6. Yu, X.-Y., Feng, Y., Guan, B., Lou, X. W. D., Paik, U. Carbon coated porous nickel phosphides nanoplates for highly efficient oxygen evolution reaction. *Energy Environ. Sci.* **9**, 1246 (2016).

7. Stern, L.-A., Feng, L. G., Song, F., Hu, X. L. Ni_2P as a Janus catalyst for water splitting: the oxygen evolution activity of Ni_2P nanoparticles. *Energy Environ. Sci.*, **8**, 2347 (2015).

8. Yang, Y., Fei, H., Ruan, G., Xiang, C., Tour, J. M. Efficient electrocatalytic oxygen evolution on amorphous nickel-cobalt binary oxide nanoporous layers. *ACS Nano* **8**, 9518 (2014).

9. Wang, H.-Y., Hsu, Y.-Y., Chen, R., Chan, T.-S., Chen, H. M., Liu, B. Ni^{3+}-induced formation of active NiOOH on the spinel Ni-Co oxide surface for efficient oxygen evolution reaction. *Adv. Energy Mater.* **5**,1500091 (2015).

10. Song, F., Hu, X. Ultrathin cobalt–manganese layered double hydroxide is an efficient oxygen evolution catalyst. *J. Am. Chem. Soc.* **136**, 16481 (2014).

11. Esswein, J., McMurdo, M. J., Ross, P. N., Bell, A. T., Tilley, T. D. Size-dependent activity of Co_3O_4 nanoparticle anodes for alkaline water electrolysis. *J. Phys. Chem. C* **113**, 15068 (2009).

12. Zhu, Y. P., Liu, Y. P., Ren, T. Z., Yuan, Z. Y. Self-supported cobalt phosphide mesoporous nanorod arrays: a flexible and bifunctional electrode for highly active electrocatalytic water reduction and oxidation. *Adv. Funct. Mater.* **25**, 7337 (2015).

13. Jin, Y. *et. al.* Porous MoO_2 nanosheets as non-noble bifunctional electrocatalysts for overall water splitting. *Adv. Mater.* **28**, 3785 (2016).

14. Sivanantham, A., Ganesan, P., Shanmugam, S. Hierarchical $NiCo_2S_4$ nanowire arrays supported on Ni foam: an efficient and durable bifunctional electrocatalyst for oxygen and hydrogen evolution reactions. *Adv. Funct. Mater.* **26**, 4661 (2016).

15. Wu, Y. Y. *et al.* Overall water splitting catalyzed efficiently by an ultrathin nanosheet-built, hollow Ni_3S_2-based electrocatalyst. *Adv. Funct. Mater.* **26**, 4839 (2016).

16. Xiao, C., Li, Y., Lu, X., Zhao, C. Bifunctional porous NiFe/$NiCo_2O_4$/Ni foam electrodes with triple hierarchy and double synergies for efficient whole cell water splitting. *Adv. Funct. Mater.* **26**, 3515 (2016).

17. Wang, X. *et. al.* Bifunctional nickel phosphide nanocatalysts supported on carbon fiber paper for highly efficient and stable overall water splitting. *Adv. Funct. Mater.* **26**, 4067 (2016).

18. Wang, H. *et. al.* Bifunctional non-noble metal oxide nanoparticle electrocatalysts through lithium-induced conversion for overall water splitting. *Nat. Commun.* **6**, 7261 (2015).

19. Jia, X., Zhao, Y., Chen, G., Shang, L., Shi, R., Kang, X., Waterhouse, G. I., Wu, L. Z., Tung, C. H., Zhang, T. Ni_3FeN nanoparticles derived from ultrathin NiFe-layered double hydroxide nanosheets: an efficient overall water splitting electrocatalyst. *Adv. Energy Mater.* **6**, 1502585 (2016).

20. Tan, Y. *et. al.* Versatile nanoporous bimetallic phosphides towards electrochemical water splitting. *Energy Environ. Sci*. **9**, 2257 (2016).

21. Tian, J. Q., Liu, Q., Asiri, A. M., Sun, X. P. Self-supported nanoporous cobalt phosphide nanowire arrays: an efficient 3D hydrogen-evolving cathode over the wide range of pH 0–14. *J. Am. Chem. Soc.* **136**, 7587 (2014).

22. Jiang, N., You, B., Sheng, M. L., Sun, Y. Electrodeposited cobalt-phosphorous-derived films as competent bifunctional catalysts for overall water splitting. *Angew. Chem. Int. Ed.* **54**, 6251 (2015).

23. Liang, Y., Liu, Q., Asiri, A. M., Sun, X., Luo, Y. Self-supported FeP nanorod arrays: a cost-effective 3D hydrogen evolution cathode with high catalytic activity. *ACS Catal.* **4**, 4065 (2014).

24. Yan, X., Tian, L., He, M., Chen, X. Three-dimensional crystalline/amorphous Co/Co_3O_4 core/shell nanosheets as efficient electrocatalysts for the hydrogen evolution reaction. *Nano.Lett.* **15**, 6015 (2015).

25. Zhang, Y., Ouyang, B., Xu, J., Chen, S., Rawat, R. S., Fan, H. J. 3D porous hierarchical nickel–molybdenum nitrides synthesized by RF plasma as highly active and stable hydrogen-evolution-reaction electrocatalysts. *Adv. Energy Mater.* **6**, 1600221 (2016).

26. Jun, H. *et. al.* Integrated three-dimensional carbon paper/carbon tubes/cobalt-sulfide sheets as an efficient electrode for overall water splitting. *ACS Nano*. **10**, 2342 (2016).

27. Wang, X. *et al.* Molybdenum phosphide as an efficient electrocatalyst for hydrogen evolution reaction. *Energy Environ. Sci.* **7**, 2624 (2014).

28. Ma, L., Ting, L. R. L., Molinari, V., Giordano, C., Yeo, B. S. Efficient hydrogen evolution reaction catalyzed by molybdenum carbide and molybdenum nitride nanocatalysts synthesized via the urea glass route. *J. Mater. Chem. A* **3**, 8361 (2015).

29. Wu, H. B., Xia, B. Y., Yu, L., Yu, X. Y., Lou, X. W. Porous molybdenum carbide nano-octahedrons synthesized via confined carburization in metal-organic frameworks for efficient hydrogen production. *Nat. Commun.* **6**, 6512 (2015).

30. Gong, M. *et al.* Nanoscale nickel oxide/nickel heterostructures for active hydrogen evolution electrocatalysis. *Nat. Commun.* **5**, 4695 (2014).

31. Vrubel, H., Hu, X. Molybdenum boride and carbide catalyze hydrogen evolution in both acidic and basic solutions. *Angew. Chem. Int. Ed.* **51**, 12703 (2012).

32. Ma, W. *et al.* A superlattice of alternately stacked Ni–Fe hydroxide nanosheets and graphene for efficient splitting of water. *ACS Nano* **9**, 1977 (2015).

33. Luo, J. *et al.* Water photolysis at 12.3% efficiency via perovskite photovoltaics and earth-abundant catalysts. *Science* **345**, 1593 (2014).

34. Jin, H. *et al. In situ* cobalt–cobalt oxide/N-doped carbon hybrids as superior bifunctional electrocatalysts for hydrogen and oxygen evolution. *J. Am. Chem. Soc.* **137**, 2688 (2015).

35. Peng, Z., Jia, D., Al-Enizi, A. M., Elzatahry, A. A., Zheng, G. From water oxidation to reduction: homologous Ni-Co based nanowires as complementary water splitting electrocatalysts. *Adv. Energy Mater.* **5**, 1402031 (2015).

36. Tang, C., Cheng, N., Pu, Z., Xing, W., Sun, X. NiSe nanowire film supported on nickel foam: an efficient and stable 3D bifunctional electrode for full water splitting. *Angew. Chem. Int. Ed. Engl.* **54**, 9351 (2015).

37. Ledendecker, M., Calderón, S. K., Papp, C., Steinrück, H.-P., Antonietti, M., Shalom, M. The synthesis of nanostructured Ni_5P_4 films and their use as a non-noble bifunctional electrocatalyst for full water splitting. *Angew. Chem. Int. Ed.* **54**, 12361 (2015).

38. Li, J.,Wang, Y., Zhou, T., Zhang, H., Sun, X.,Tang, J., Zhang, L., Al-Enizi, A. M.,Yang, Z., Zheng, G. Nanoparticle superlattices as efficient bifunctional electrocatalysts for water splitting. *J. Am. Chem. Soc*. **137**, 14305 (2015).

39. Hou, Y. *et al.* Vertically oriented cobalt selenide/NiFe layered-double-hydroxide nanosheets supported on exfoliated graphene foil: an efficient 3D electrode for overall water splitting. *Energy Environ. Sci.* **9**, 478 (2016).

40. Liu, D. N., Lu, Q., Luo, Y. L., Sun, X. P., Asiri, A. M. $NiCo_2S_4$ nanowires array as an efficient bifunctional electrocatalyst for full water splitting with superior activity. *Nanoscale*, **7**, 15122 (2015).

41. Liang, H., Li, L., Meng, F., Dang, L., Zhuo, J., Forticaux, A., Wang, Z., Jin, S. Porous two-dimensional nanosheets converted from layered double hydroxides and their applications in electrocatalytic water splitting. *Chem. Mater.* **27**, 5702 (2015).

42. Lai, J., Li, S., Wu, F., Saqib, M., Luque, R., Xu, G. Unprecedented metal-free 3D porous carbonaceous electrodes for full water splitting. *Energy Environ. Sci.* **9**, 1210 (2016).

43. McCrory, C., Jung, S., Peters, J. C., Jaramillo, T. Benchmarking heterogeneous electrocatalysts for the oxygen evolution reaction. *J. Am. Chem. Soc.* **135**, 16977 (2013).
