# Supplementary material for: Value added transformation of ubiquitous substrates into highly efficient and flexible electrodes for water splitting
Source: Nat Commun. 2018 May 22;9:2014. doi: 10.1038/s41467-018-04358-7 (PMC5964234; doi:10.1038/s41467-018-04358-7)
Supplement: Supplementary file 3 — Description of Additional Supplementary Files [file 41467_2018_4358_MOESM3_ESM.pdf]

Description of Additional Supplementary Files

File Name: Supplementary Movie 1
Description: NiFe/Ni-P paper as working electrode at an operating potential of 1.6V v/s RHE in 1M KOH.

File Name: Supplementary Movie 2
Description: NiMo/Ni-P paper as working electrode at an operating potential of -0.1V v/s RHE in 1M KOH.

File Name: Supplementary Movie 3
Description: NiFe-NiMo 2-electrode alkaline electrolyzer at a cell voltage of 1.85V in 1M KOH.
